# Supplementary material for: Recurrent Viral Capture of Cellular Phosphodiesterases that Antagonize OAS-RNase L
Source: bioRxiv. 2023 Sep 14:2023.05.12.540623. Preprint. [Version 2] doi: 10.1101/2023.05.12.540623 (PMC10515750; doi:10.1101/2023.05.12.540623)
Supplement: Supplement 1 [file media-1.pdf]

A

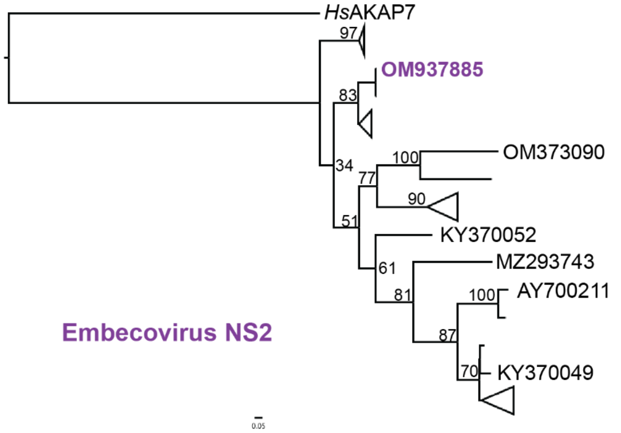

B

Reference: OM937885

Virus	RdRp % identity	PDE % identity	Core PDE % identity
OM373090	86.3	47.2	47.6
KY370052	94	61.1	60.8
MZ293743	91.4	50.5	50
AY700211	90.3	51.5	51.6
KY370049	91.4	51.5	51.3

C

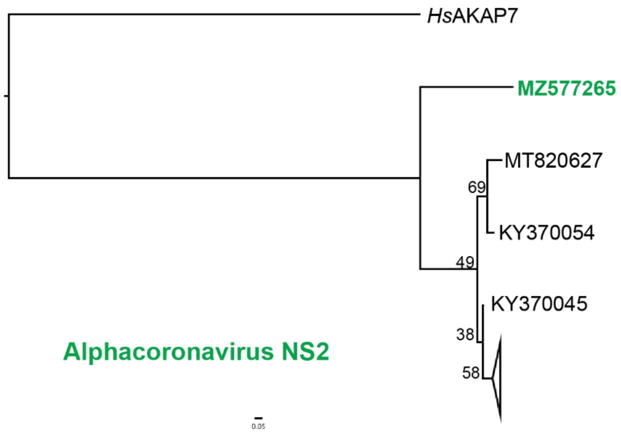

D

Reference: MZ577265

Virus	RdRp % identity	PDE % identity	Core PDE % identity
MT820627	93.4	53.4	54.4
KY370054	93.1	55.9	57.7
KY370045	94	55.9	55.5

E

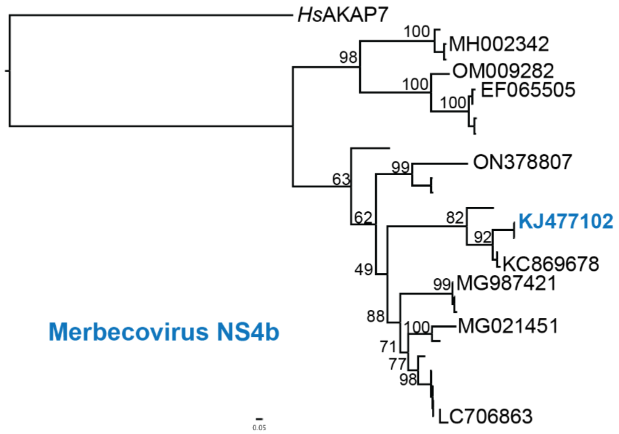

F

Reference: KJ477102

Virus	RdRp % identity	PDE % identity	Core PDE % identity
MH002342	92.1	25.9	30.4
OM009282	89.8	27.3	29
EF065505	90	27	28.8
ON378807	94.5	35.3	44.5
KC869678	98.4	83.7	90.5
MG987421	95	40.3	45.7
MG021451	94.3	39.7	48.6
LC786603	94.6	41.6	48.6

G

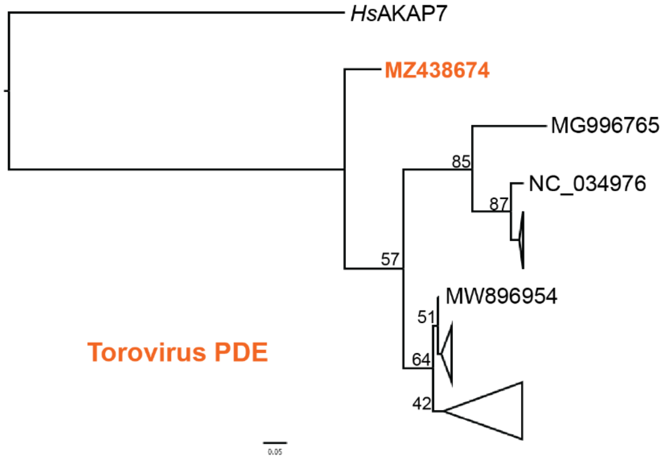

H

Reference: MZ438674

Virus	RdRp % identity	PDE % identity
MG996765	83.5	71.6
NC_034976	85.5	70.2
MW896954	89.3	78.7

I

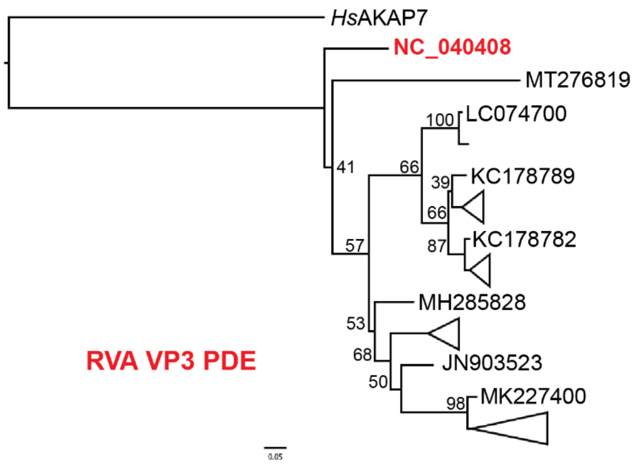

J

Reference: NC_040408

Virus	VP3 % identity	PDE % identity
MT276819	71.6	66.9
LC074700	74.6	73.2
KC178789	74.1	72.5
KC178782	75.4	71.1
MH285828	76.8	77.5
JN903523	75.8	72.5
MK227400	76.5	73.2

K

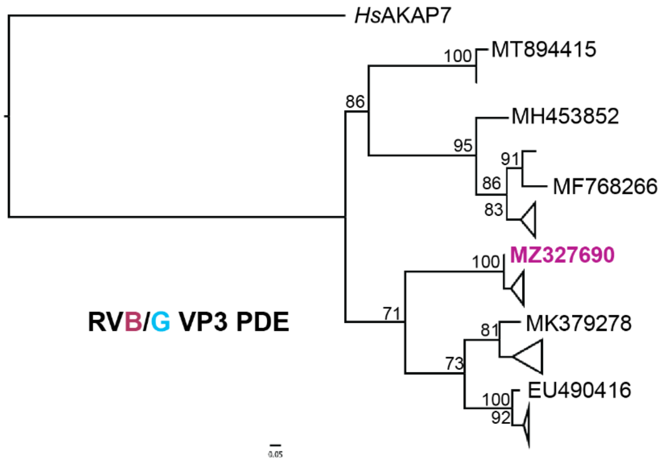

L

Reference: MZ327690

Virus	VP3 % identity	PDE % identity
MT894415	49.7	50.4
MH453852	44.1	44.3
MF768266	46.1	47
MK379278	62.5	53.9
EU490416	63.6	54.8

Figure S1. PDEs encoded by discrete open reading frames exhibit higher sequence divergence than sub-domain PDEs A) ML tree of 55 Embecovirus NS2 AA sequences rooted with hAKAP7 PDE and with the

reference sequence for AA identity comparisons colored and bolded B) Table of AA identity of select Embecovirus RdRp, PDE, and ‘Core’ PDE sequences to the reference sequence. C) ML tree of 10 Alphacoronavirus NS2 AA sequences rooted with hAKAP7 PDE and with the reference sequence for AA identity comparisons colored and bolded D) Table of AA identity of select Alphacoronavirus RdRp, PDE, and ‘Core’ PDE sequences to the reference sequence. E) ML tree of 27 Merbecovirus NS4b AA sequences rooted with hAKAP7 PDE and with the reference sequence for AA identity comparisons colored and bolded. F) Table of AA identity of select Merbecovirus RdRp, PDE, and ‘Core’ PDE sequences to the reference sequence. G) ML tree of 18 Torovirus PDE AA sequences rooted with hAKAP7 PDE and with the reference sequence for AA identity comparisons colored and bolded. H) Table of AA identity of select Torovirus RdRp, and PDE sequences to the reference sequence. I) ML tree of 35 Rotavirus A PDE rooted with hAKAP7 and with the reference sequence for AA identity comparisons colored and bolded. J) Table of AA identity of select Rotavirus A PDE and VP3 (minus the PDE) sequences to the reference sequence. K) ML tree of 20 Rotavirus B and 8 Rotavirus G PDE sequences rooted with hAKAP7 and with the reference sequence for AA identity comparisons colored and bolded. L) Table of AA identity of select Rotavirus B and G PDE and VP3 (minus the PDE) sequences to the reference sequence.

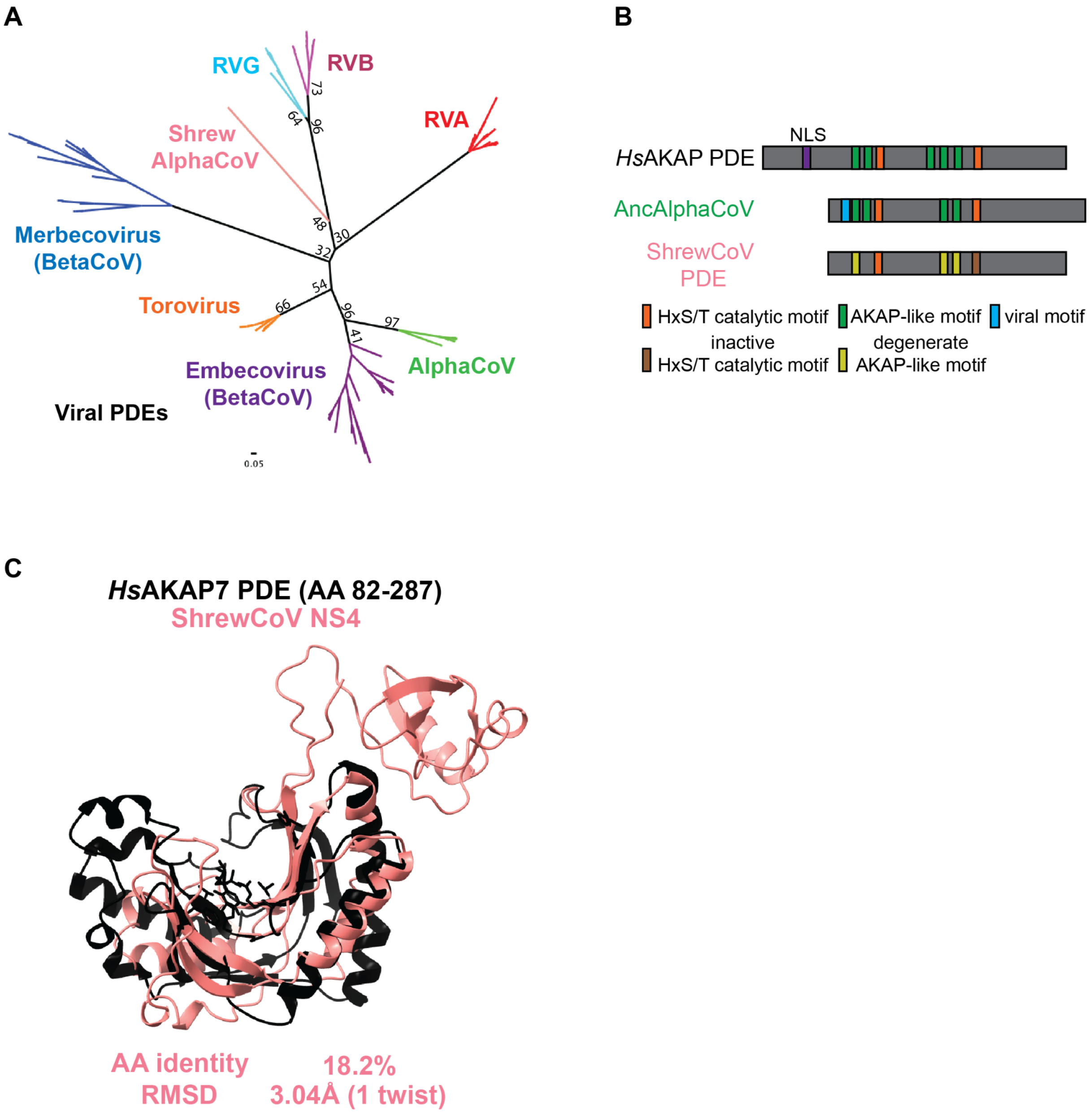

Figure S3. Phylogenetic and structural characterization of divergent, non-functional ShrewCoV PDE. A) ML tree of all virus PDEs, including the NS4 protein of ShrewCoV. B) Schematic of the ShrewCoV PDE in comparison to the *Hs*AKAP7 and rodent AlphaCoV PDEs, showing intact and degenerate but identifiable motifs. C) Overlay and RMSD comparison of *Hs*AKAP7 PDE and ShrewCoV NS4.

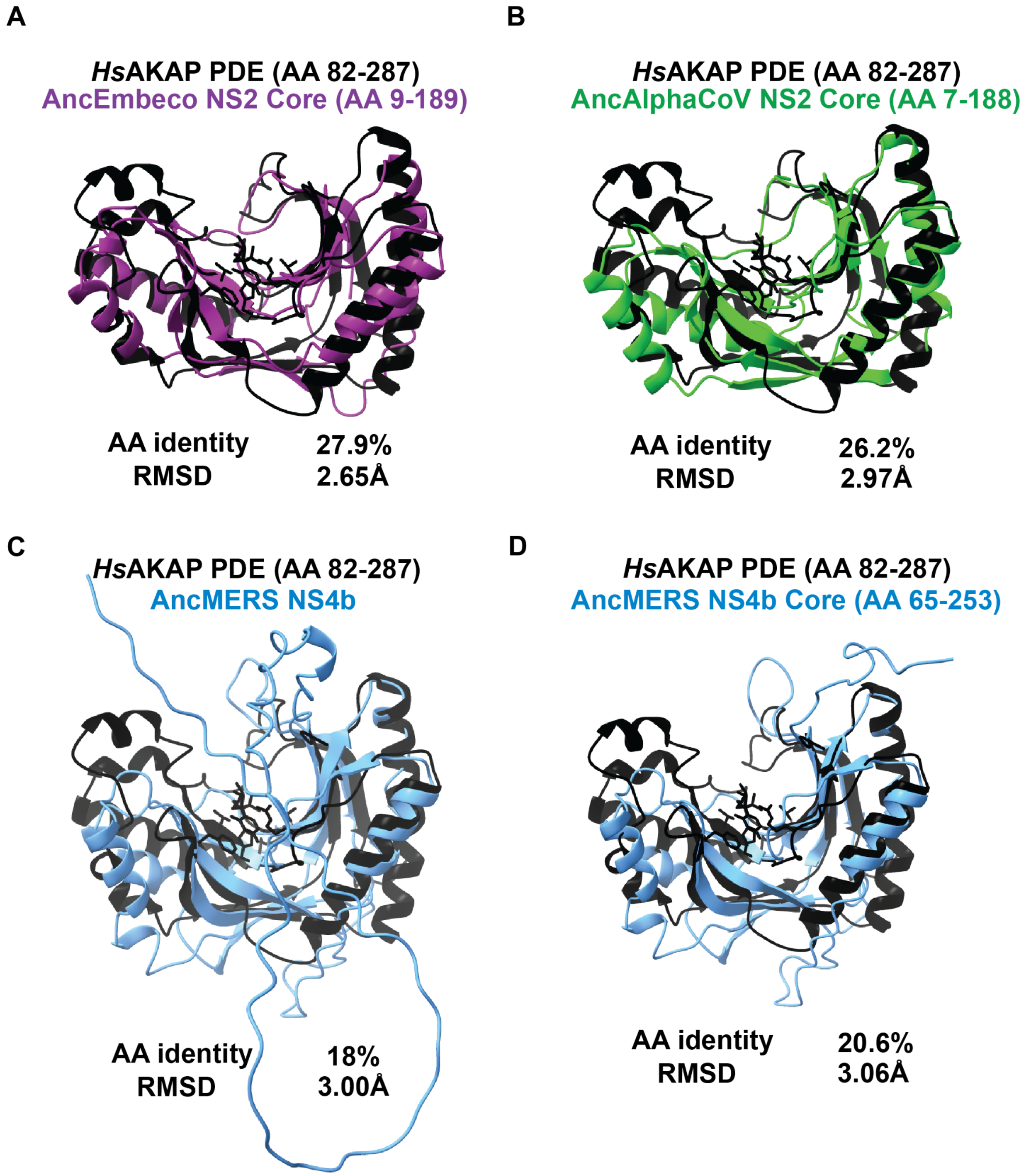

Figure S4. Sequence and structural divergence is not due to unique N and C termini of viral PDEs. A) Sequence and structural comparison of hAKAP PDE and the AncEmbeco NS2 core PDE predicted structure. B) Sequence and structural comparison of hAKAP PDE and the AncAlphaCoV NS2 core PDE predicted structure. C) Sequence and structural comparison of hAKAP PDE and the AncMERS NS4b PDE predicted structure. The AncMERS PDE was chosen because AlphaFold failed to predict a structure for the AncMerbeco core PDE domain D) Sequence and structural comparison of hAKAP PDE and the AncMERS NS4b core PDE predicted structure.
