# Supplementary material for: Recurrent Viral Capture of Cellular Phosphodiesterases that Antagonize OAS-RNase L
Source: bioRxiv. 2023 Sep 14:2023.05.12.540623. Preprint. [Version 2] doi: 10.1101/2023.05.12.540623 (PMC10515750; doi:10.1101/2023.05.12.540623)
Supplement: 1 [file NIHPP2023.05.12.540623V2-supplement-1.pdf]

bioRxiv preprint doi: https://doi.org/10.1101/2023.05.12.540623; this version posted September 14, 2023. The copyright holder for this preprint (which was not certified by peer review) is the author/funder, who has granted bioRxiv a license to display the preprint in perpetuity. It is made available under aCC-BY 4.0 International license.

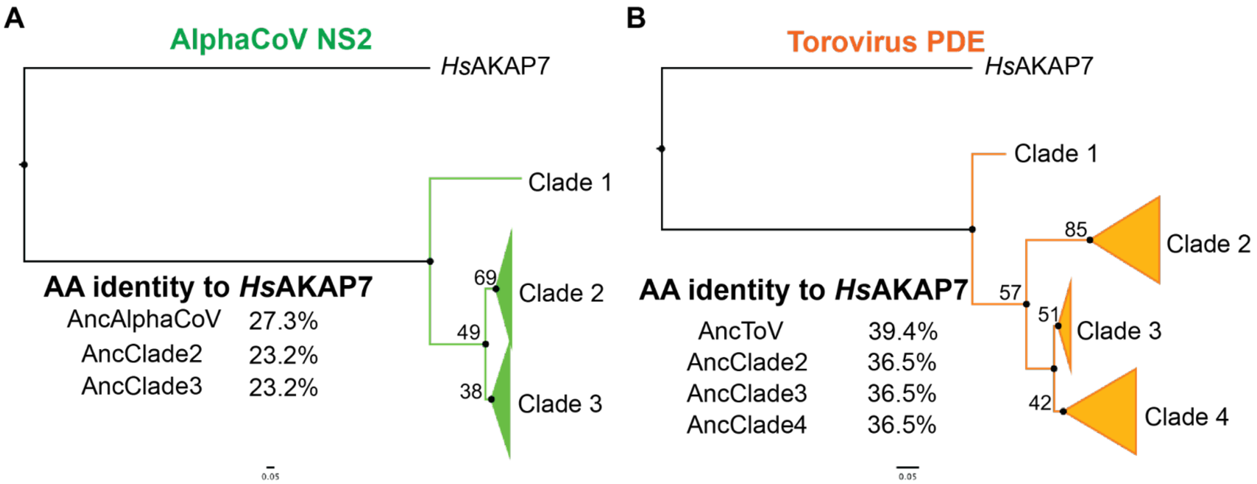

Figure S2. Ancestral sequence reconstruction (ASR) of Alphacoronavirus and Torovirus PDEs. A) ML tree of 10 Alphacoronavirus PDE sequences, collapsed into major clades, rooted with the *Hs*AKAP7 PDE domain sequence (AA 82-287) and sequence identity to *Hs*AKAP7 of reconstructed sequences at major nodes. B) ML tree of 18 Torovirus PDE sequences, collapsed into major clades, rooted with the *Hs*AKAP7 PDE domain sequence (AA 82-287) and sequence identity to *Hs*AKAP7 of reconstructed sequences at major nodes.
